# Evidence of past forest fragmentation in the Congo Basin from the phylogeography of a shade-tolerant tree with limited seed dispersal: *Scorodophloeus zenkeri* (Fabaceae, Detarioideae)

**DOI:** 10.1186/s12862-021-01781-1

**Published:** 2021-03-30

**Authors:** Samuel Vanden Abeele, Steven B. Janssens, Rosalía Piñeiro, Olivier J. Hardy

**Affiliations:** 1 Meise Botanic Garden, Nieuwelaan 38, 1860 Meise, Belgium; 2 Evolutionary Biology and Ecology, Faculté Des Sciences, Université Libre de Bruxelles, Av. F.D. Roosevelt 50, 1050 Brussels, Belgium; 3 Plant Conservation and Population Biology, KU, Leuven, Belgium; 4 Geography, College of Life and Environmental Sciences, University of Exeter, Laver Building, North Park Road, Exeter, EX4 4QE UK; 5 Present Address: School of Integrative Plant Sciences, Section of Plant Biology and the L.H. Bailey Hortorium, Cornell University, Ithaca, NY 14853 USA

**Keywords:** Phylogeography, Population genetics, Tropical Africa, Rainforest, Gene dispersal, Spatial genetic structure, Selfing, Glacial forest refugia

## Abstract

**Background:**

Comparative phylogeographic studies on rainforest species that are widespread in Central Africa often reveal genetic discontinuities within and between biogeographic regions, indicating (historical) barriers to gene flow, possibly due to repeated and/or long-lasting population fragmentation during glacial periods according to the forest refuge hypothesis. The impact of forest fragmentation seems to be modulated by the ecological amplitude and dispersal capacities of each species, resulting in different demographic histories. Moreover, while multiple studies investigated the western part of Central Africa (Lower Guinea), few have sufficiently sampled the heart of the Congo Basin (Congolia). In this study, we look for genetic discontinuities between populations of the widespread tropical tree *Scorodophloeus zenkeri* Harms (Fabaceae, Detarioideae) in Central Africa. Additionally, we characterize genetic diversity, selfing rate and fine-scale spatial genetic structure within populations to estimate the gene dispersal capacity of the species.

**Results:**

Clear intraspecific genetic discontinuities occur throughout the species’ distribution range, with two genetic clusters in Congolia and four in Lower Guinea, and highest differentiation occurring between these bioregions. Genetic diversity is higher in Lower Guinea than Congolia. A spatial genetic structure characteristic of isolation by distance occurs within the genetic clusters. This allowed us to estimate gene dispersal distances (σ_g_) for this outcrossing species with ballistic seed dispersal, which range between 100 and 250 m in areas where *S. zenkeri* occurs in high densities, and are in the low range of σ_g_ values compared to other tropical trees. Gene dispersal distances are larger in low density populations, probably due to extensive pollen dispersal capacity.

**Conclusions:**

Fragmentation of *S. zenkeri* populations seems to have occurred not only in Lower Guinea but also in the Congo Basin, though not necessarily according to previously postulated forest refuge areas. The lower genetic diversity in Congolia compared to Lower Guinea parallels the known gradient of species diversity, possibly reflecting a stronger impact of past climate changes on the forest cover in Congolia. Despite its bisexual flowers, *S. zenkeri* appears to be mostly outcrossing. The limited dispersal observed in this species implies that genetic discontinuities resulting from past forest fragmentation can persist for a long time before being erased by gene flow.

**Supplementary Information:**

The online version contains supplementary material available at 10.1186/s12862-021-01781-1.

## Background

The rainforest in West and Central Africa, known as the Guineo–Congolian rainforest, is renowned for its high species richness and endemism rate, harbouring more than 10,000 vascular plant species of which more than 30% are endemic [1, 2]. Based on species distribution ranges and endemism rates, different biogeographic units can be recognised; Upper Guinea (UG) in West Africa, Lower Guinea (LG) in Atlantic Central Africa and Congolia in the Congo Basin [1, 3, 4]. Although Congolia represents the largest of the three subregions [1, 5], its diversity in terms of number of plant species is the lowest (approx. 3,900 species, 6% endemic) and it remains poorly explored. By contrast, Lower Guinea is the most diverse (approx. 7,000 species, 24% endemic) [1] and best explored to date [2, 6].

Although the distribution and diversity of biogeographic regions is often studied at the species level or higher taxonomical level, additional insights can be gained by investigating differences in intraspecific genetic diversity within those bioregions (i.e., phylogeography). Indeed, diversity patterns at the level of communities (species richness) and populations (genetic diversity) are partially driven by similar processes (ecological and genetic drift, migration, community and population sizes fluctuations [7]), generating similar signatures of past biogeographic events (e.g., range fragmentation or expansion). Since molecular dating shows that congeneric plant species in tropical Africa often diverged before the Pleistocene (2.6 Myr ago) [5, 8–11], the impact of recent climatological events on the African bioregions can best be observed when studying patterns of genetic diversity at the intraspecific level. Comparative phylogeographic studies that span multiple biogeographic regions often reveal genetic differentiation between bioregions, thereby giving an indication of putative (historical) barriers to gene flow [5, 8, 10, 12–17]. In addition, many of the investigated tree species display differentiated gene pools within Lower Guinea. This indicates the occurrence of repeated or long-lasting population fragmentation, hence supporting the Pleistocene forest refuge hypothesis driven by climatic oscillations in the case of rainforest trees, shrubs and liana species. Like species richness, patterns of genetic differentiation have mostly been studied and reported for Lower Guinea, while little is known about the structuring of genetic variation and the impact of past climatic fluctuations in Congolia. Previously postulated refuge hypotheses of lowland rainforest [18, 19] show important discrepancies for Congolia. While Maley [19] hypothesised the occurrence of a continuous large refuge in Congolia based on gradients of species richness, Anhuf et al. [18] postulated a scenario of highly fragmented refugia based on estimates of past rainfall. Furthermore, the response of tropical tree species to forest fragmentation appears to be modulated by different demographic histories, possibly depending on the species’ ecological amplitude and dispersal capacities [5].

In the current study, we aim to assess the impact of past forest fragmentation events in Central Africa, with a special focus on Congolia, by investigating putative genetic discontinuities in the widespread tropical African tree species *Scorodophloeus zenkeri* Harms (Fabaceae, Detarioideae). *Scorodophloeus zenkeri* is a medium-sized to large tree (up to 40 m in height) and is a typical element of mixed evergreen forests with well-drained soils [20]. As a result, the demographic history of the species is expected to be closely linked to that of lowland rainforests. The species is absent from West Africa and widely distributed in Lower Guinea and the Congo Basin, including Cameroon, Equatorial Guinea, Gabon, Republic of the Congo (abbr. Congo), Cabinda (Angola) and the Democratic Republic of the Congo (abbr. DR Congo). *Scorodophloeus zenkeri* is typical for dense mature evergreen forests and since it does not tolerate waterlogging, the species is absent in the northern part of Congo. This causes a gap in its distribution area, meaning that populations from Lower Guinea and Congolia are currently only connected in the south-western part of DR Congo. *Scorodophloeus zenkeri* is a shade-tolerant tree that can be dominant in mature *terra firme* rainforests (which are for instance found in inland Gabon and around Kisangani in DR Congo), and it often has an aggregated distribution [15, 20]. The species is characterized by a ballochorous type of seed dispersal, i.e., through explosion of the fruit pods, which suggests a limited seed dispersal capacity [12, 15].

By applying genetic clustering methods on nuclear microsatellite data from *S. zenkeri*, we evaluate the impact of past forest fragmentation in Central African lowland rainforests. Furthermore, genetic diversity indices are calculated to identify regions with a high intraspecific diversity (indicative of ancient forest refugia), and genetic divergence is estimated. In addition, we study the fine-scale spatial genetic structure in populations throughout the species’ distribution range, as well as seed dispersal distances in order to assess if the dispersal capacity of this species is limited, as suggested by the fruit morphology.

Within the current study, the following questions are addressed: (1) Are the populations from Lower Guinea and Congolia genetically differentiated (i.e., are there genetic clusters unique to Congolia)? (2) If so, do we observe genetic discontinuities between populations within Congolia, indicating (ancient) population fragmentation in forest refugia? (3) How efficient is gene dispersal in *S. zenkeri*?

## Results

### Are *Scorodophloeus zenkeri* populations characterized by genetic discontinuities?

**Fig. 1:**
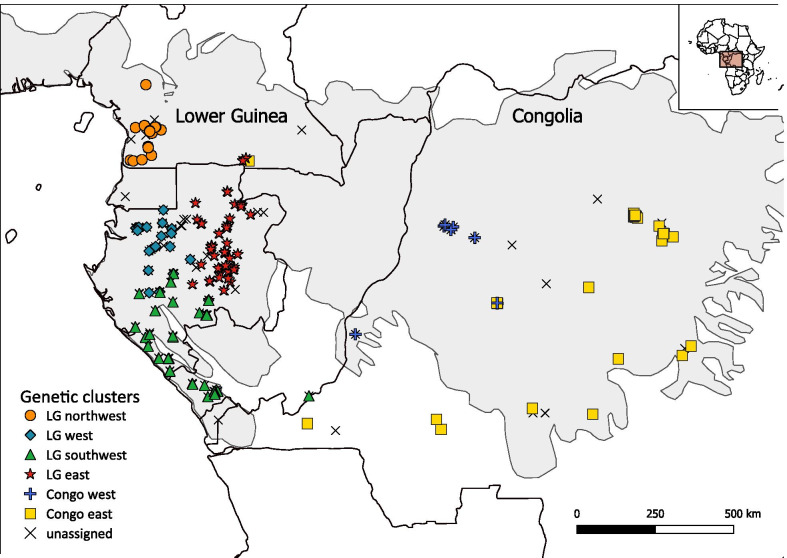
Distribution of genetic clusters in *Scorodophloeus zenkeri* as inferred with STRUCTURE for the most likely scenario at *K* = 6. The grey area depicts the natural distribution of rainforests in Central Africa. The map was made using QGIS 3.4 [22]

As described below (Methods section), different clustering analyses and an ordination of genotypic data revealed that the *S. zenkeri* populations in Lower Guinea are genetically well differentiated from those in Congolia, with both biogeographic regions harbouring several distinct genetic subclusters (four in Lower Guinea and two in Congolia; Fig. 1).

The Bayesian clustering algorithm implemented in STRUCTURE [21] inferred five to six genetic clusters, depending on the dataset and parameters used, as well as the criterion used to identify the optimal number of clusters. Using the complete SSR dataset and the same STRUCTURE parameters as in Piñeiro et al. [12], the average log-likelihood of the data Ln *P*(*D*) substantially increased up to *K* = 3, followed by a small increase up to *K* = 6, and a small decrease to *K* = 10. The run with the highest Ln *P*(*D*) was observed at *K* = 6, as well as the highest average Ln *P*(*D*) among the 10 iterations (Additional file 1: Figure S1A). Variability in Ln *P*(*D*) among iterations was low across all *K*-values.

**Fig. 2:**
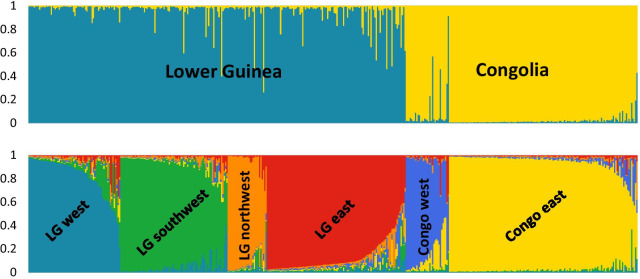
The bar plots for *K* = 2 and *K* = 6 representing the assignment probabilities (vertical axis) inferred using STRUCTURE*,* for the complete *S. zenkeri* SSR1 dataset (*n* = 465)

Mapping of the six putative clusters in QGIS (Fig. 1) showed four clusters that are unique to Lower Guinea and corresponded to the ones described by Piñeiro et al. [12]; one in the southwestern part of Cameroon (north western Lower Guinea, hereafter referred to as ‘LG northwest’), in north western Gabon (referred to as ‘LG west’), in eastern Gabon (referred to as ‘LG east’), and one in the southwestern part of the distribution range (referred to as ‘LG southwest’). In addition, two clusters were unique to Congolia; one in western DR Congo (referred to as ‘Congo west’) and one covering the southern and eastern area (referred to as ‘Congo east’). In STRUCTURE, populations from Lower Guinea were first separated from the Congolian populations (at *K* = 2) (Fig. 2). Subsequently, the major LG cluster was further subdivided at higher *K*-values (*K* = 3 to 5) (Additional file 1: Figure S2). Lastly, the major Congolian cluster was further subdivided (at *K* = 6) (Fig. 2).

Using a subsampled SSR dataset in STRUCTURE (keeping ≤ 3 samples per km^2^ to avoid bias due to overrepresented areas [23]), the average Ln *P*(*D*) increased with *K*, first substantially up to *K* = 3, then slightly up to *K* = 5 (Additional file 1: Figure S1B). The run with the highest Ln *P*(*D*) was observed at *K* = 5, as well as the highest average Ln *P*(*D*) among the 10 iterations. At *K* = 5, the same clusters obtained with the complete SSR dataset were retrieved, except that the two Congolian clusters under *K* = 6 were grouped into a single cluster. To further investigate this discrepancy, we ran an additional analysis in STRUCTURE restricted to samples from Congolia (i.e., samples with longitude > 16° east, after subsampling to avoid biased clustering due to overrepresentation of certain localities,* n* = 95). This additional clustering run indicated *K* = 2 as most plausible solution (Additional file 1: Figure S3A), supporting the subdivision of the Congolian populations, though 19% of the samples showed admixed genotypes with assignment probabilities between 0.2 and 0.8 (Additional file 1: Figure S3B).

The maximum-likelihood genetic clustering method implemented in the *snapclust* function of the *adegenet* [24] package in R [25] showed that the best supported solution was *K* = 7 when the complete SSR dataset was used (Additional file 1: Figure S4A), and* K* = 6 using the subsampled SSR dataset (Additional file 1: Figure S4B). The *K* = 7 solution was similar to that obtained using STRUCTURE, except that cluster ‘LG east’ was further subdivided into two geographically overlapping clusters. Hence, this solution seemed suboptimal. However, the solution at *K* = 6 had a similar support and the geographic distribution of these six clusters approximately matched the distribution of the STRUCTURE clusters (Additional file 1: Figure S5). Some geographic anomalies were found, mostly corresponding to admixed samples that were classified as ‘unassigned’ in the STRUCTURE analysis.

Hence, we opted for *K* = 6 as the most plausible number of genetic clusters in our SSR dataset, since congruence with geography was good as well. Compared to the study by Piñeiro et al. [12], which was mostly focussed on Lower Guinea, current analysis revealed an additional cluster in Congolia, while the same four genetic clusters were inferred in Lower Guinea, even with the inclusion of 85 new samples from that bioregion. All the inferred genetic clusters showed a parapatric or allopatric distribution, with unassigned/admixed individuals (*n* = 99, assignment probability *q* < 0.8) mostly occurring in contact zones between genetic clusters (Fig. 1).

In the ordination of genotypes by PCA (Additional file 1: Figure S6), the first axis (4.24% of variance explained) separated Congolian clusters from Lower Guinean clusters, axis 2 (2.89% var. expl.) separated ‘LG east’ from the other LG clusters, axis 3 (2.14% var. expl.) mostly separated ‘LG west’ from the other LG clusters, and axis 4 (1.73% var. expl.) partly separated clusters ‘LG northwest’ and ‘LG west’. Unassigned/admixed individuals occupied intermediate positions between genetic clusters.

### Are populations in Lower Guinea more genetically diverse than those in Congolia?

The diversity analyses revealed that the *S. zenkeri* populations in Lower Guinea are characterized by a higher genetic diversity than those in Congolia (Table 1). The effective number of alleles (*NAe*) and the observed heterozygosity (*Ho*) were highest in cluster ‘Congo west’, while the allelic richness (*AR*) was highest in cluster ‘LG southwest’ (Table 1). Genetic diversity was lowest in the cluster ‘LG northwest’ (*NAe* = 2.32, *AR* = 4.53 and *Ho* = 0.36), followed by ‘Congo east’ (*NAe* = 2.71, *AR* = 4.42 and *Ho* = 0.48). Estimated inbreeding (*Fi*) was significantly different from 0 in all clusters except for ‘Congo west’, which is most probably due to the presence of null alleles (as null alleles were observed for six out of ten loci according to STRUCTURE results, data not shown) and potentially biparental inbreeding. *Scorodophloeus zenkeri* has bisexual flowers but it appears to be an outcrossing species, since the estimated selfing rate was non-significantly different from zero for all genetic clusters (Table 1).

**Table 1:** Genetic diversity parameters of the six inferred genetic clusters and for the main bioregions (Lower Guinea and Congolia) in *Scorodophloeus zenkeri* populations

Cluster	*n*	*NAe*	*AR* (k = 26)	*He*	*Ho*	*Fi*	*Sp *(± *SE*)	*SR *(± *SE*)
LG northwest	22	2.32	4.53	0.43	0.36	0.16	/	0 ± 0.144
LG east	83	3.58	5.36	0.61	0.50	0.18	0.010 ± 0.002	0.001 ± 0.047
LG west	46	3.20	5.22	0.63	0.49	0.23	0.013 ± 0.005	0.112 ± 0.123
LG southwest	66	3.24	5.49	0.55	0.48	0.13	0.009 ± 0.002	0.050 ± 0.088
Congo west	18	3.87	4.83	0.58	0.54	0.07	/	0 ± 0
Congo east	131	2.71	4.42	0.52	0.48	0.09	0.003 ± 0.001	0 ± 0.006
*All clusters*	*465*	*4.20*	*6.29*	*0.68*	*0.49*	*0.28*	/	/

Bioregion	*n*	*NAe*	*AR* (k = 126)	*He*	*Ho*	*Fi*		
Lower Guinea	204	3.99	10.06	0.66	0.47	0.29		
Congolia	76	3.22	6.51	0.58	0.49	0.16		

*n* number of individuals analysed, *NAe* effective number of alleles [26], *AR (k* = x*)* allelic richness or number of alleles among x gene copies, *He* expected heterozygosity, *Ho* observed heterozygosity, *Fi* individual inbreeding coefficient, *Sp* statistic used to quantify the decay of kinship-distance curves within the best represented clusters (minimum *n* = 46) based on the regression slope of pairwise kinship coefficients on the logarithm of geographic distances [27], *SR* selfing rate estimate [28], *SE* standard error. *Fi* was significantly larger than 0 for all clusters, except for ‘Congo west’. The selfing rate estimate was non-significant for all genetic clusters

Pairwise genetic differentiation as measured by *F*_*ST*_ ranged from 0.12 (‘LG west’ vs ‘LG southwest’) to 0.30 (‘LG northwest’ vs ‘Congo west’) and is generally lower (*F*_ST_ < 0.2) between clusters from the same bioregion (i.e., within Lower Guinea or Congolia), except for cluster ‘LG northwest’ which is well differentiated from all the other ones, and ‘Congo west’ which is not much differentiated from ‘LG east’ (*F*_ST_ = 0.16) (Table 2). When accounting for allele size, differentiation as measured by *R*_*ST*_ showed similar results, with very low differentiation between the two Congolian clusters (*R*_*ST*_ = 0.06). Allele size permutation tests showed no significant difference between *F*_*ST*_ and *R*_*ST*_ for any pair of clusters, indicating that differentiation between clusters results more from genetic drift than the accumulation of stepwise mutations [29], which suggests that the clusters diverged less than 1/*µ* generations ago, where µ is the mutation rate of microsatellites [29]. Assuming that *µ* ranges between 10^–4^ and 10^–3^, and that the generation time of *S. zenkeri* is between 100 and 200 years [16, 30], we expect clusters to have diverged less than 2 Ma ago, thus during the Quaternary, as also supported by Piñeiro et al. [12] using demographic simulations.

**Table 2:** Genetic differentiation between the six inferred genetic clusters in *Scorodophloeus zenkeri* as measured by *F*_ST_ (below diagonal) and *R*_ST_ (above diagonal)

*F*_ST_\*R*_ST_	LG northwest	LG east	LG west	LG southwest	Congo west	Congo east
LG northwest		0.17	0.20	0.23	0.25	0.15
LG east	0.20		0.16	0.23	0.16	0.14
LG west	0.24	0.14		0.26	0.30	0.24
LG southwest	0.21	0.18	0.12		0.33	0.29
Congo west	0.30	0.16	0.23	0.26		0.06
Congo east	0.28	0.23	0.26	0.25	0.17	

Allele size permutation tests detected no significant shift in allele size (*R*_ST_ > *F*_ST_) between any pair of clusters

### How efficient is gene dispersal?

**Fig. 3:**
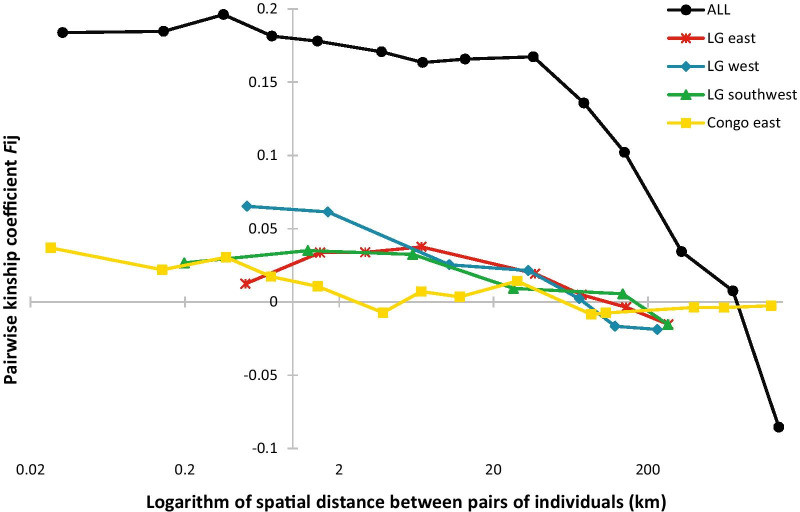
Kinship-distance curves for the pairs of individuals (*q* ≥ 0.8) according to log(distance) within the largest genetic clusters (*n* > 46; ‘LG east’, ‘LG west, ‘LG southwest’ and ‘Congo east’) and for pairs of individuals from the complete SSR dataset (‘ALL’). For the largest genetic clusters, decaying curves illustrate isolation by distance patterns (trend of decay of kinship *Fij* with distance). For the complete SSR dataset (‘ALL’), the decline of *Fij* at distances smaller than 50 km is the result of isolation by distance within each genetic cluster, while the steep decline of *Fij* at distances larger than 50 km probably reflects genetic divergence between genetic clusters as a result of ancient population fragmentation, since an increasing proportion of pairs of individuals belong to distinct genetic clusters with increasing distances

When assessing the spatial genetic structure in the complete SSR dataset, the kinship coefficient (*F*_*ij*_) first decayed slowly from 0.18 to 0.16 between individuals separated by less than 1 km to 50 km, then decayed quickly at larger distances, reaching negative values beyond c. 800 km (i.e., samples more than 800 km apart are on average less related than two random individuals from our sampling; Fig. 3). The resulting *Sp* statistic was 0.058 (Table 1). In contrast, within the four best-represented clusters the kinship-distance curves decayed slowly, starting at values smaller than 0.065 at short distances and never reaching very negative values, leading to statistically significant *Sp* statistics ranging from 0.003 to 0.013 (Fig. 3; Table 1). The observed patterns indicate that most of the spatial genetic structure observed at the continental scale results from the differentiation between the six genetic clusters, most probably caused by barriers to gene flow during historic population fragmentation. The spatial genetic structuring observed within each cluster is most likely the result of isolation by distance. Assuming the latter, we estimated gene dispersal distance (σ_g_) using a method implemented in SPAGeDi [31] based on the relationship between the slope of the kinship-distance curve, the effective population density (*De*) and σ_g_ under an isolation by distance theoretical model [27].

The kinship-distance decay measured in the more densely sampled areas of the Yangambi and Yoko reserves, within cluster ‘Congo east’, led to *Sp* = 0.0051 ± 0.0013 and to indirect estimates of gene dispersal distances reaching σ_g_ = 116 m, 166 m and 228 m (Additional file 1: Table S1) when considering the regression between σ_g_ and 20σ_g_, and assuming an effective density equal to ½, ¼ and 1/10 of the density of adult trees (*D* = 18 ha^−1^), respectively. Similar gene dispersal distance estimates (σ_g_ = 105 m, 154 m and 241 m) were obtained when considering the regression between σ_g_ and 100σ_g_. Cluster ‘LG northwest’ was characterized by the lowest density of adult trees (*D* = 0.14 ha^−1^) and gene dispersal distance estimates were much higher, reaching 1861 to 2334 m (Additional file 1: Table S1). At intermediate densities, gene dispersal estimates ranged between 299 and 533 m in cluster ‘LG west’ (*D* = 1.35 ha^−1^), and between 394 and 1136 m in cluster ‘LG southwest’ (*D* = 1.12 ha^−1^), illustrating the negative correlation between gene dispersal distance and population density. No estimates were obtained for ‘LG east’ since the iterative method in SPAGeDi did not converge. This could be because the genetic structure in this cluster does not reflect isolation by distance or because the distribution of genotyped samples was not optimal for such inference. In contrast to σ_g_ estimates, neighbourhood size estimates were quite stable, ranging between 125 and 134 in the Yangambi and Yoko reserves, between 105 and 152 in the cluster ‘LG northwest’, between 48 and 76 in ‘LG west’, and between 90 and 182 in ‘LG southwest’.

## Discussion

The tropical tree *Scorodophloeus zenkeri* shows clear intraspecific genetic discontinuities throughout its Central African distribution range. Our clustering analyses revealed that the populations in Lower Guinea are genetically well differentiated from those in Congolia, with each bioregion harbouring several distinct genetic clusters. While Congolia is underrepresented in previous phylogeographic studies, our study is among the first to indicate that forest fragmentation also occurred in this bioregion. The division of the Lower Guinean and Congolian bioregions [3, 4], which is based on the distribution of endemic species, seems to be supported by the distribution of intraspecific genetic diversity within *S. zenkeri*. Additionally, the overall premise that Lower Guinea has a higher diversity and rate of endemism than Congolia in terms of species diversity [1], is also confirmed at the intraspecific level for *S. zenkeri* in terms of allelic diversity (Table 1), a pattern also occurring in the tree *Parkia bicolor* [32]. The current study is the first to document this differential distribution of intraspecific genetic diversity in plants, which could be explained by a more drastic reduction of the forest cover in Congolia compared to Lower Guinea. In such a scenario, the occurrence of multiple forest refugia in Lower Guinea would have enabled the preservation of the genetic diversity in stable populations, while more genetic diversity would have been lost inland in Congolia [13, 15, 33]. Furthermore, Lower Guinea is characterized by a more heterogeneous landscape (elevation variability and savannah-forest mosaics), thereby enhancing differentiation between stable populations in forest refugia and inducing genetic diversity [13, 15].

In Lower Guinea, our dataset revealed the same four genetic clusters as inferred by Piñeiro et al. based on a reduced sampling [12, 34]. As documented for several other woody and herbaceous species in Lower Guinea [5, 12–15], genetic differentiation appears to be strongest between northern (‘LG northwest’) and southern populations. Such congruent genetic discontinuities observed in multiple unrelated plant species support a scenario of ancient population fragmentation, followed by recolonization.

In Congolia, two distinct genetic clusters were inferred for *S. zenkeri*, thereby suggesting that the glacial periods during the Pleistocene also caused fragmentation of the Congolian rainforest. Reconstruction of the rainforest distribution area at the time of the Last Glacial Maximum (LGM), based on past rainfall distribution patterns, suggested the occurrence of multiple forest refugia fragments in Congolia [18]. In contrast, based on the current gradients of species richness and endemism, a single large rainforest refugium has been inferred for Congolia (covering large parts of the Congo and Kasaï rivers) [19]. The two distinct genetic clusters detected in our study support the presence of multiple historical forest refuge fragments. Similarly, based on climatic niche models, two relatively small LGM climatic refugia were inferred for *S. zenkeri* in Congolia [34]. However, none of the postulated refuge areas coincide with the genetic clusters inferred in the present study. Such discrepancies between postulated refuge areas and current distribution of intraspecific diversity were also observed in Lower Guinea on both shade-tolerant and pioneer tree species [5, 12, 15, 35]. These discrepancies between intraspecific genetic clusters and postulated forest refuge areas could be caused by the very limited data on which the LGM refugia in Congolia were inferred by Anhuf et al. [18] and Maley [19]. Also, the multiplicity of forest fragmentation events proceeding the LGM did not necessarily lead to the same forest fragments at each glacial maximum. Since molecular dating indicates that Central African tree populations often diverged before the LGM [8, 36], genetic discontinuities could be linked to one or multiple older glaciation events.

The open-forest formations and swamp forests that dominate northeastern Republic of the Congo [37] seem to coincide with the genetic break between *S. zenkeri* populations in Lower Guinea and Congolia. This occurrence of open-forest formations with an abundance of light demanding tree species has been linked to past human activities and drier climatic periods during the Holocene [15]. It is possible that populations from north-eastern Gabon, northern Congo and north-western DR Congo were not completely isolated during Pleistocene forest retractions (persistent gene flow through micro-refugia such as gallery forest along rivers for example), explaining the moderate level of differentiation between these Lower Guinean and Congolian genetic clusters (*F*_ST_ = 0.16), and that the present-day distribution break solely originates from the Holocene vegetation changes. However, since the divergence between genetic clusters within Lower Guinea was linked to forest retractions during the Pleistocene [12], the main split between Lower Guinea and Congolia likely predates the Holocene. In this scenario, the moderate level of genetic differentiation between ‘LG east’ and ‘Congo west’ could be the result of secondary gene flow between both regions after long-term isolation during the Pleistocene.

Gene dispersal estimates revealed limited dispersal capacities of *S. zenkeri,* in agreement with its gregarious distribution and a fruit morphology that suggests ballistic dispersal [12, 20]. Our spatial genetic analyses show that the spatial genetic structure within the different clusters as quantified by the *Sp* statistic (0.009 to 0.013 in LG, 0.003 in Congolia) lies in the range of *Sp* values found in other tropical tree species, but is lower than the average of 0.017 [38]. However, the spatial genetic structure resulting from isolation by distance depends on both gene dispersal distances and population density. When considering the later within a densely sampled area where the density of *S. zenkeri* appears fairly high (c. 18 adult trees per ha), gene dispersal distances (σ_g_) due to seed and pollen dispersal appears to be limited, with estimates of σ_g_ ranging from 105 to 241 m. This is in the low range of estimates reported for tropical trees, where σ_g_ typically ranges from a few hundred meters [39–42] to a few kilometres [43, 44]. Hence, our results confirm that gene dispersal distances are very limited in areas where *S. zenkeri* occurs at high densities. While limited seed dispersal was expected, our results suggest that pollen dispersal must be quite limited as well, at least in those high-density areas such as the Yangambi and Yoko forests. In areas where *S. zenkeri* occurs at lower density, such as in northwestern and southwestern Lower Guinea, gene dispersal distances appear to be much higher, with estimates up to c. 2300 m. This inverse correlation between population density and gene dispersal distance is expected since pollen dispersal is more extensive under low population density simply because potential mating pairs are more distant from each other [17, 39, 40]. Indeed, the fact that chloroplast DNA markers show a higher divergence for populations in southwestern Gabon [12, 15], while nuclear microsatellites infer only one genetic cluster for this area, could be explained if seed dispersal is more restricted than pollen dispersal in *S. zenkeri*. Southwestern Gabon is characterized by a highly heterogeneous landscape with a lot of elevation variability and savannah-forest mosaics, and *S. zenkeri* is uncommon here [15]. Since chloroplast DNA is generally maternally inherited, the distribution of chloroplast genes usually reflects seed dispersal. Therefore, the high divergence observed from the chloroplast markers implies that the savannah areas which surround suitable rainforest habitat might constitute a barrier for seed dispersal, while pollen (which contribute to the dispersal of nuclear genes) could ensure population connectivity at a large scale.

Despite the current continuous distribution of *S. zenkeri* in Congolia and Lower Guinea, the historical genetic structure has not been erased (yet). This can be explained by the long generation time of tropical trees such as *S. zenkeri*, meaning that millions of years might be needed to homogenize populations through gene flow [8, 12, 15]. Additionally, *S. zenkeri* is a shade-tolerant tree, indicative of mature forests, and incapable of colonizing open habitats. This, in combination with its limited dispersal capacities, can explain its slow spatial dynamics and the preservation of the ancient genetic structure induced by biogeographic events.

## Conclusions

The present study reveals intraspecific genetic discontinuities within the tropical tree *Scorodophloeus zenkeri* throughout Central Africa, caused by ancient population fragmentation, presumably during glacial periods. The populations in Lower Guinea are genetically differentiated from those in Congolia, with each bioregion harbouring distinct genetic clusters. While Congolia is underrepresented in previous phylogeographic studies, our study suggests that forest fragmentation also occurred in this bioregion, though not necessarily according to previously postulated refuge hypotheses. The lower genetic diversity in Congolia compared to Lower Guinea possibly reflects a stronger impact of past climate changes on the forest cover. The fine-scale genetic structure detected in *S. zenkeri* populations confirms that seed and pollen dispersal are very limited, at least in the dense populations of eastern Congo, further limiting gene flow among genetic clusters, even though *S. zenkeri* is mostly outcrossing. In low density populations, gene dispersal distances are larger, since pollen dispersal is forced to be more extensive. Future phylogeographic studies will increasingly apply whole-genome datasets, therewith enabling accurate dating of population divergence, and providing a more detailed image of the evolutionary history of the Central African rainforest.

## Methods

### Sampling, DNA isolation and genotyping

Silica-dried leaf samples or cambium slashes were collected from 174 *Scorodophloeus zenkeri* trees on field missions in Cameroon, Gabon, Congo and DR Congo. Additionally, leaf material was collected from 91 dried herbarium specimens present in the herbarium of Meise Botanic Garden (BR). Total DNA was extracted from the 265 individuals using a modified cetyltrimethylammonium bromide (CTAB) protocol [45] that included an additional sorbitol washing step.

Ten nuclear microsatellite loci were amplified in *S. zenkeri* using two multiplex reactions as described by Piñeiro et al. [12]. Fragment analysis was done on an ABI 3730 DNA Analyzer (Applied Biosystems) with 1 µL PCR product, 12µL Hi-Di Formamide (Applied Biosystems) and 0.3 µL GeneScan 500 LIZ dye (Applied Biosystems) as size standard. The newly generated electropherograms were combined with electropherograms from 240 individuals previously genotyped at the same loci [12] and mostly originating from Lower Guinea. To ensure that genotypes of both datasets were read in the same way, peak calling and delimitation of bins was done for all 505 samples together, using the Microsatellite Plugin 1.4.6 in Geneious 9.1.8 (Biomatters Ltd.). Only samples for which at least six out of ten loci amplified, were used in subsequent analyses. Pairwise relationship coefficients [46] were calculated using SPAGeDi 1.5d [31] to check for duplicated individuals. If such duplicates were identified, samples were removed so that only one sample per individual tree remained. The combined dataset (*n* = 465) generated in this study includes 174 samples from the Congo Basin, making it the first phylogeographic study on *S. zenkeri* to completely cover the known species’ distribution area (Fig. 1) [47].

### Genetic clustering

Genetic clusters were first inferred using the Bayesian clustering algorithm implemented in the STRUCTURE software 2.3.4 [21]. Two runs were done to assess the impact of the sampling scheme. First, the complete SSR dataset (*n* = 465) was run using the same parameters as described by Piñeiro et al. [12]: admixture model, independent allele frequencies, number of clusters set between *K* = 1 and *K* = 10, and 10 iterations for each *K*. Both the burn-in period and the number of MCMC replicates were set at 100,000. Second, the same settings were used for running a reduced SSR dataset (*n* = 368), which was obtained by subsampling a maximum of three individuals per square area with a side of 0.01° (approximately 1 km^2^), since some areas were more densely sampled (e.g., the Yangambi and Yoko reserves in DR Congo). This was done to test whether such an overrepresentation of geographic areas was causing a biased clustering [23]. For both clustering runs in STRUCTURE, recessive null alleles were declared for each locus, so that null allele frequencies were directly estimated and accounted for. To determine the most optimal number of clusters *K*, the log-likelihood of the data Ln *P*(*D)* was plotted against *K* [21] and the stability of replicate runs for each *K* was assessed.

To ensure a robust clustering outcome, genetic clusters were also inferred using the *snapclust* function of the *adegenet* [24] package in R [25], which applies an expectation–maximization (EM) algorithm for maximum-likelihood based clustering. The maximum number of putative clusters was set to 10.

The genetic clusters inferred with both methods were mapped using QGIS 3.4.5 to assess their geographic distribution. Genetic diversity in the complete SSR dataset was summarized with a principal component analysis (PCA) and visualized as a scatterplot with R [25] packages *adegenet* [24] and *ade4* [48].

### Diversity indices and spatial genetic structuring

After removing admixed individuals (highest assignment probability *q* < 0.8) from the inferred genetic clusters, the following multilocus diversity parameters were calculated with SPAGeDi 1.5d [31]: expected heterozygosity (*He*), observed heterozygosity (*Ho*), rarefied allelic richness (*AR*), effective number of alleles (*NAe*), and inbreeding coefficient (*F*IS). The same genetic diversity parameters were calculated for Lower Guinea and Congolia, after subsampling to avoid bias due to overrepresented areas. Furthermore, *F*_ST_ [49] and *R*_ST_ [50] were calculated to assess pairwise genetic differentiation between inferred clusters. As *R*_ST_ is based on allele size, it can be used to estimate the contribution of stepwise mutations to genetic differentiation [29]. Therefore, observed *R*_ST_ values were compared to the *F*_ST_ values obtained from 10,000 permutations of allele sizes among alleles, to test for a phylogeographic signal at the SSRs.

Patterns of spatial genetic structure were analysed in the complete dataset and within the largest inferred genetic clusters (minimum *n* = 46) by calculating the pairwise kinship coefficient (*F*_*ij*_) between individuals from the respective clusters, and plotting *F*_*ij*_ against the geographic distance between compared individuals. The decay of the kinship-distance curves was quantified by the *Sp* statistic, based on the regression slope of *F*_*ij*_ on the logarithm of geographic distances [27]. Significance of the slope was assessed by comparing observed *F*_*ij*_ to their frequency distributions obtained after 10,000 random permutations of all individuals (or those assigned to the respective clusters) with respect to their spatial positions [51].

### Estimating gene dispersal and selfing rate

We estimated gene dispersal distances from the kinship-distance decay in the more densely sampled clusters (all but ‘Congo west’, see ‘Results’ section), using an indirect method based on isolation-by-distance models [27, 39]. This method relies on the theoretical expectation that *F*_*ij*_ decays approximately linearly with ln(distance) at a rate proportional to (*Fn*-1)/(4π.*De*. σ_g_^2^), where σ_g_^2^ is half the mean squared parent–offspring distance, *De* the effective density of adults, and *Fn* the mean *F*_*ij*_ between neighbours, here considered as individuals separated by less than 100 m. This theoretical approximation is good for distances ranging from σ_g_ to approximately 0.56σ_g_/(2µ)^1/2^, where µ is the mutation rate [52]. Therefore, the regression slope was computed for distances larger than σ_g_ and lower than 20σ_g_ or 100σ_g_, while σ_g_ was estimated using an iterative procedure implemented in SPAGeDi 1.5d [31], where *De* must be given as a fixed parameter. Using 100σ_g_ as upper limit for the regression allowed us to increase precision by considering more *F*_*ij*_ pairs, but at the cost of some upward bias if µ > 10^–5^. We assumed that *De* ranged between one tenth and one half of the density of adult trees [39]. For cluster ‘Congo east’, dispersal distances were estimated in the more densely sampled Yangambi and Yoko reserves. Forest inventories indicate a *S. zenkeri* density per hectare of 23 trees with a diameter at breast height (dbh) larger than 10 cm, and 14 trees with a dbh larger than 30 cm in the Yoko reserve (400 ha inventoried, F. Boyemba, pers. comm.), and of 35 trees with a dbh larger than 10 cm in the Yangambi reserve (11 ha inventoried, H. Beeckman, pers. comm.). Assuming that *S. zenkeri* reproduces regularly from a dbh of 30 cm, and has similar dbh structures at both localities, we considered a mean density of 18 adults per ha. Thus, we tested the estimation procedure of σ_g_ assuming that *De* equals 9, 4.5 or 1.8 individuals per ha (i.e., ½, ¼ and 1/10^th^ of the adult density). Large-scale forest inventories in Lower Guinea ([53], Réjou-Méchain, pers. comm.) indicate the following *S. zenkeri* densities (dbh > 30 cm): 0.14 trees/ha for ‘LG northwest’ (688 ha inventoried), 4.51 trees/ha for ‘LG east’ (4402 ha inventoried), 1.35 trees/ha for ‘LG west’ (4764 ha inventoried), and 1.12 trees/ha for ‘LG southwest’ (6366 ha inventoried).

Finally, selfing rate was estimated in each genetic cluster. In order to avoid bias due to null alleles, selfing was estimated from identity disequilibrium, with standard errors estimated by jackknifing over loci [28, 54]. All calculations were done using SPAGeDi 1.5d [31].

## Supplementary Information

**Additional file 1.** Additional figures and tables.

## Data Availability

The datasets used and analysed during the current study are available from the corresponding author on reasonable request.

## Abbreviations

UG: Upper Guinea
LG: Lower Guinea
Myr: Million years ago
Congo: Republic of the Congo
DR Congo: Democratic Republic of the Congo
SSR: Simple sequence repeat
PCA: Principal component analysis
Ma: Million years ago
LGM: Last Glacial Maximum
dbh: Diameter at breast hight
