# Supplementary material for: Evidence of past forest fragmentation in the Congo Basin from the phylogeography of a shade-tolerant tree with limited seed dispersal: Scorodophloeus zenkeri (Fabaceae, Detarioideae)
Source: BMC Ecol Evol. 2021 Mar 30;21:50. doi: 10.1186/s12862-021-01781-1 (PMC8011194; doi:10.1186/s12862-021-01781-1)
Supplement: Supplementary file 1 — Additional file 1. Additional figures and tables. [file 12862_2021_1781_MOESM1_ESM.docx]

Vanden Abeele et al. 2021 – Supplementary material

**(A)**


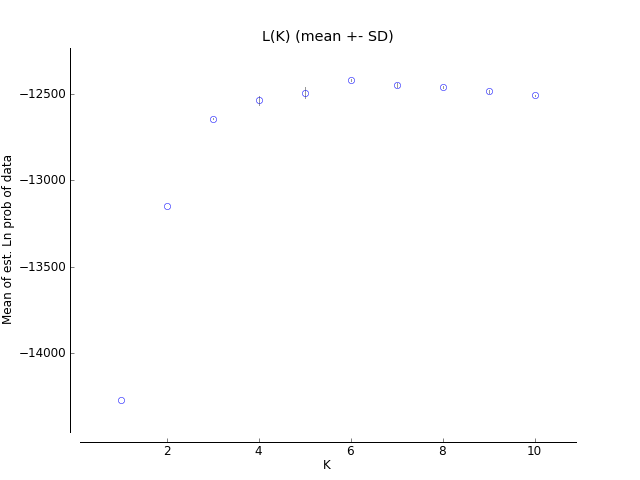


**(B)**


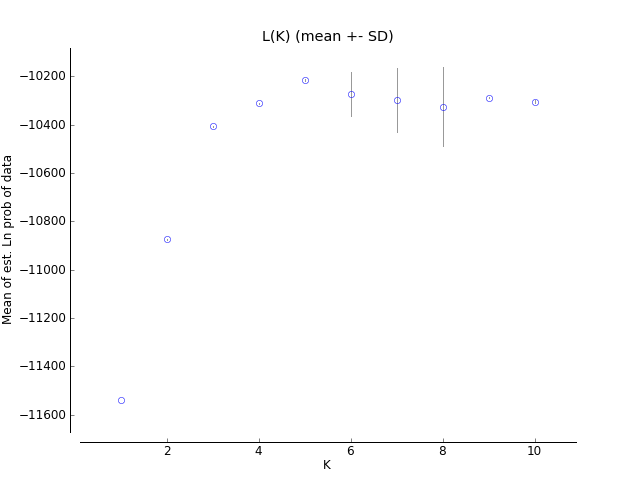


**Suppl. Fig. 1:** Likelihood of the *Scorodophloeus zenkeri* SSR dataset as a function of the assumed number of genetic clusters (*K*) according to the Bayesian clustering algorithm implemented in STRUCTURE, using the **admixture model and the independent allele frequencies** model as in a previous study on *Scorodophloeus zenkeri* [1]. Two different datasets were used to assess the impact of overrepresented geographical areas [2]. **(A)** The **complete SSR dataset** (*n* = 465) or **(B)** the **subsampled SSR dataset** (*n* = 368) obtained by subsampling a maximum of three individuals per square area with a side of 0.01° (approximately 1 km²). The circle represents the mean log-likelihood over 10 runs, while the vertical bars show the standard deviation among runs.

**Lower Guinea**

**Congolia**

**Congo east**

**Congo west**

**LG northwest**

**LG west**

**LG east**

**LG southwest**

**Suppl. Fig. 2:** The bar plots for *K* = 2 to *K* = 6 representing the assignment probabilities (y-axis) inferred using STRUCTURE*,* for the complete *Scorodophloeus zenkeri* SSR1 dataset (*n* = 465).

**(A)**


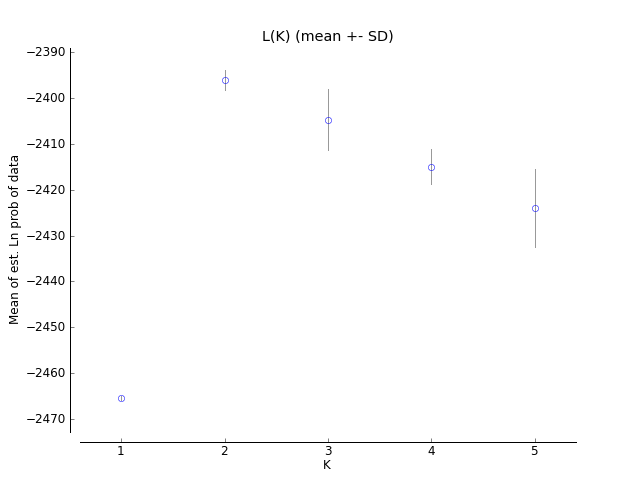


**(B)**

**Suppl. Fig. 3: (A)** Likelihood of the *Scorodophloeus zenkeri* SSR dataset, only including samples from Congolia (i.e. with longitude > 16° east, *n* = 95), as a function of the assumed number of genetic clusters (*K*) according to the Bayesian clustering algorithm implemented in STRUCTURE, using the same parameters as in a previous study on *Scorodophloeus zenkeri* [1]. **(B)** The bar plot for *K* = 2 representing the assignment probabilities inferred using STRUCTURE.

**(A)**


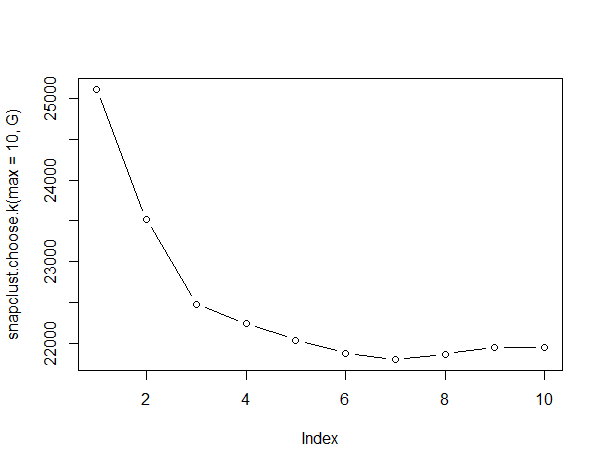


**(B)**


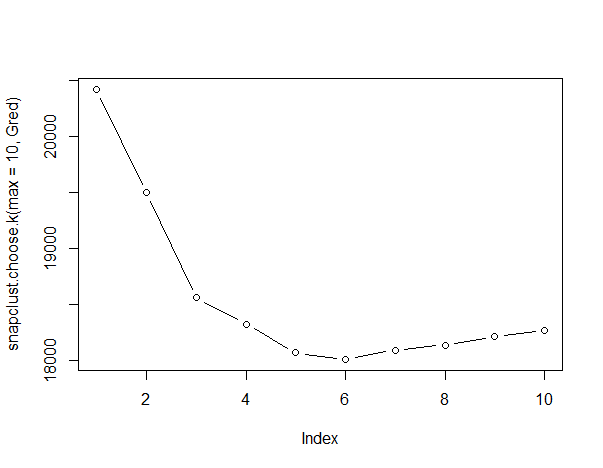


**Suppl. Fig. 4:** Likelihood of the *Scorodophloeus zenkeri* SSR dataset as a function of the assumed number of genetic clusters (*index*), using an expectation-maximization (EM) algorithm for maximum-likelihood based clustering implemented in the *snapclust* function of the *adegenet* [3] package in R [4]. Two different datasets were used to assess the impact of overrepresented geographical areas [2]. **(A)** The **complete datase**t (SSR1, *n* = 465) or **(B)** the **subsampled dataset** (SSR2, *n* = 368) obtained by subsampling a maximum of three individuals per square area with a side of 0.01° (approximately 1 km²).


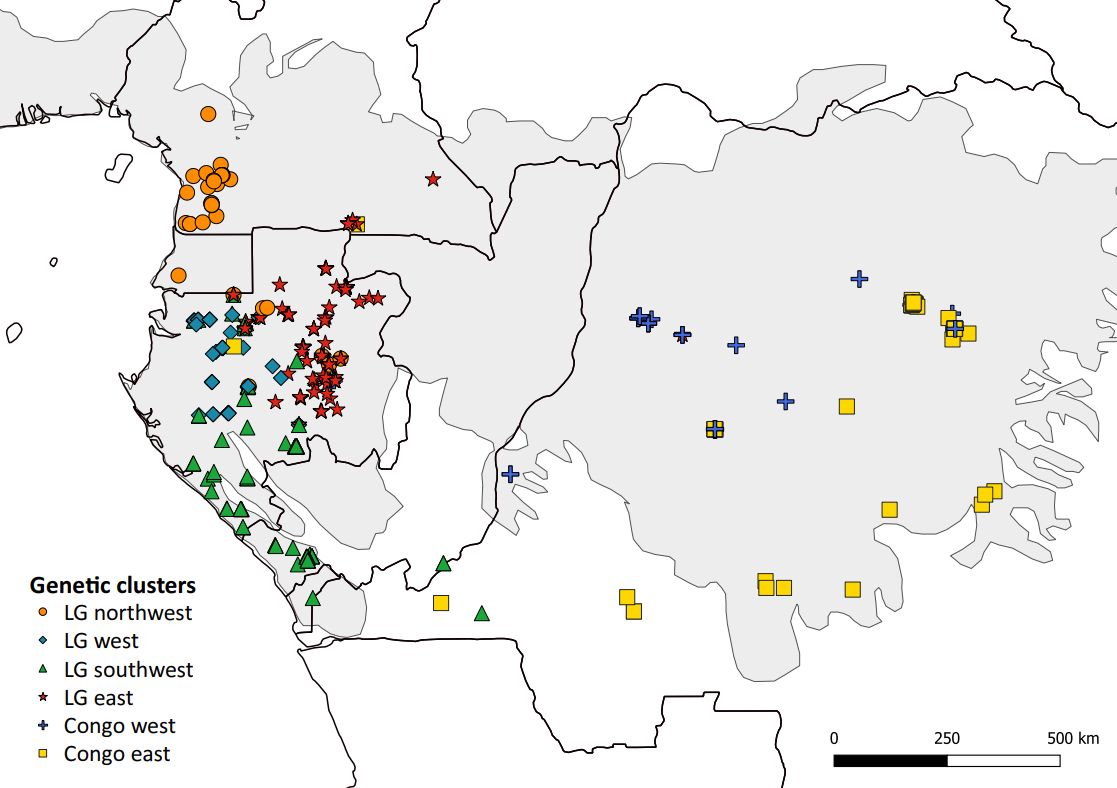


**Suppl. Fig. 5:** Distribution of genetic clusters in *Scorodophloeus zenkeri* as inferred with the *snapclust* function of the R package *adegenet*, for the most likely scenario at *K* = 6. The grey area depicts the natural distribution of rainforests in Central Africa. The map was made using QGIS 3.4 [5].


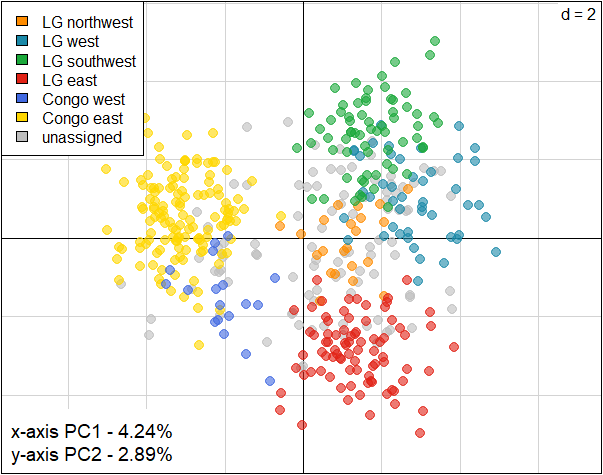


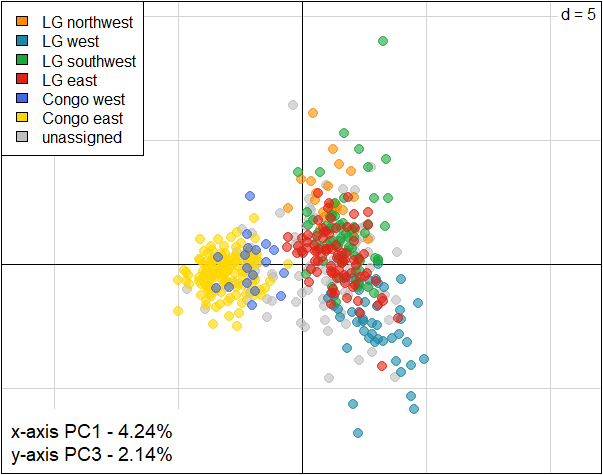


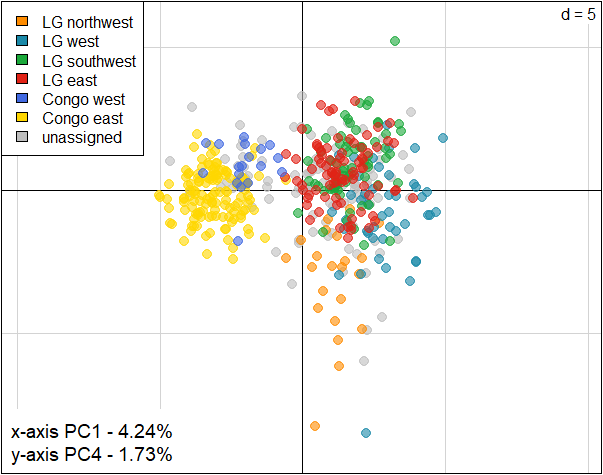


**Suppl. Fig. 6:** Scatterplots visualizing the Principal Component Analysis (PCA) of the genetic diversity in the *Scorodophloeus zenkeri* SSR dataset. Groups assignments of the individuals (coloured dots) correspond to the genetic clusters inferred with STRUCTURE at *K* = 6 (assignment probability *q* ≥ 0.8). Axes are rescaled in each scatterplot to allow the visualization of all data points (*d* = width between gridlines). *PC* stands for Principal Component.

**Suppl. Table 1:** Gene dispersal distance (σ_g_) and neighbourhood size (*Nb*) estimates for the best represented genetic clusters and localities, using three estimates of effective densities (*De* = *D*/2, *D*/4 and *D*/10) and considering the regression between σ_g_ and 100 σ_g_. *na* indicates that the iterative method did not converge. The standard error by jacknifing over loci (*SE*) is given unless convergence was not achieved.

| Cluster/locality | *n* | *De* | Trees/km² | σ_g_ (m) (± *SE*) | *Nb* (± *SE*) |
| --- | --- | --- | --- | --- | --- |
| LG northwest | 22 | *D*/2 | 7 | *na* | *na* |
|  |  | *D*/4 | 3.5 | 1,861 | 152 |
|  |  | *D*/10 | 1.4 | 2,334 | 105 |
| LG east | 83 | *D*/2 | 225.5 | *na* | *na* |
|  |  | *D*/4 | 112.8 | *na* | *na* |
|  |  | *D*/10 | 45.1 | *na* | *na* |
| LG west | 46 | *D*/2 | 67.5 | 299 (± 51) | 76 (± 26) |
|  |  | *D*/4 | 33.8 | 358 (± 102) | 54 (± 34) |
|  |  | *D*/10 | 13.5 | 533 (± 245) | 48 (± 51) |
| LG southwest | 66 | *D*/2 | 56 | 394 (± 42) | 113 (± 23) |
|  |  | *D*/4 | 28 | 505 (± 181) | 90 (± 69) |
|  |  | *D*/10 | 11.2 | 1,136 (± 251) | 182 (± 89) |
| Yangambi & Yoko | 113 | *D*/2 | 900 | 105 (± 41) | 125 (± 106) |
| (Congo east) |  | *D*/4 | 450 | 154 (± 53) | 134 (± 97) |
|  |  | *D*/10 | 180 | 241 (± 92) | 132 (± 108) |

**References**

1. Piñeiro R, Dauby G, Kaymak E, Hardy OJ. Pleistocene population expansions of shade-tolerant trees indicate fragmentation of the African rainforest during the Ice Ages. Proc R Soc B Biol Sci. 2017;284:20171800. doi:10.1098/rspb.2017.1800.

2. Puechmaille SJ. The program structure does not reliably recover the correct population structure when sampling is uneven: Subsampling and new estimators alleviate the problem. Mol Ecol Resour. 2016;16:608–27.

3. Jombart T. adegenet: a R package for multivariate analysis of genetic markers. Bioinformatics. 2008;24:1403–5.

4. R Development Core Team. R: A language and environment for statistical computing. 2011.

5. QGIS.org. QGIS Geographic Information System. 2021. https://www.qgis.org/.
